# L-Glutamine Supplementation Enhances Strength and Power of Knee Muscles and Improves Glycemia Control and Plasma Redox Balance in Exercising Elderly Women

**DOI:** 10.3390/nu13031025

**Published:** 2021-03-22

**Authors:** Gislene R. Amirato, Juliana O. Borges, Daniella L. Marques, Juliana M. B. Santos, Carlos A. F. Santos, Marilia S. Andrade, Guilherme E. Furtado, Marcelo Rossi, Lais N. Luis, Raquel F. Zambonatto, Eliane B. da Silva, Sarah O. Poma, Mariana M. de Almeida, Renato L. Pelaquim, Laiane C. dos Santos-Oliveira, Vinicius L. Sousa Diniz, Maria E. P. Passos, Adriana C. Levada-Pires, Renata Gorjão, Marcelo P. Barros, André L. L. Bachi, Tania C. Pithon-Curi

**Affiliations:** 1 Interdisciplinary Post-Graduate Program in Health Sciences, Institute of Physical Activity Sciences and Sports (ICAFE), Cruzeiro do Sul University, São Paulo, SP 01506-000, Brazil; prof.gislene@hotmail.com (G.R.A.); julili_borges@hotmail.com (J.O.B.); daniella.m.lima.dl@gmail.com (D.L.M.); laisnascimento_pa@hotmail.com (L.N.L.); zambonattoraquel@gmail.com (R.F.Z.); eliane_borges91@hotmail.com (E.B.d.S.); sarah.poma@hotmail.com (S.O.P.); maribonnes@gmail.com (M.M.d.A.); renato.pelaquim@gmail.com (R.L.P.); laiane.cris@hotmail.com (L.C.d.S.-O.); viniciusleo_diniz@hotmail.com (V.L.S.D.); melizabethpp@gmail.com (M.E.P.P.); adriana.pires@cruzeirodosul.edu.br (A.C.L.-P.); renata.gorjao@cruzeirodosul.edu.br (R.G.); tania.pithon-curi@cruzeirodosul.edu.br (T.C.P.-C.); 2 Post-Graduation Program in Science of Human and Rehabilitation, Federal University of São Paulo (UNIFESP), Santos, SP 11015-020, Brazil; juliana-mbs@hotmail.com; 3 Department of Medicine (Geriatrics and Gerontology), Federal University of São Paulo (UNIFESP), São Paulo, SP 04020-050, Brazil; freitas.carlos@uol.com.br; 4 Department of Physiology, Federal University of São Paulo (UNIFESP), São Paulo, SP 04023-901, Brazil; marilia.andrade@unifesp.br; 5 Health Sciences Research Unit: Nursing (UICISA:E), Nursing School of Coimbra (ESEnfC), 3000-232 Coimbra, Portugal; guilhermefurtado@esenfc.pt; 6 Post-Graduation Program in Health Sciences, Santo Amaro University (UNISA), São Paulo, SP 04829-300, Brazil; mrossi@dim.fm.usp.br (M.R.); allbachi77@gmail.com (A.L.L.B.); 7 ENT Lab, Department of Otorhinolaryngology, Federal University of São Paulo (UNIFESP), São Paulo, SP 04023-062, Brazil

**Keywords:** physical exercise, aging, oxidative stress, antioxidant, muscle contraction performance, diabetes, sarcopenia

## Abstract

We investigated the effects of oral L-glutamine (Gln) supplementation, associated or not with physical exercises, in control of glycemia, oxidative stress, and strength/power of knee muscles in elderly women. Physically active (*n* = 21) and sedentary (*n* = 23) elderly women aged 60 to 80 years were enrolled in the study. Plasma levels of D-fructosamine, insulin, reduced (GSH) and oxidized (GSSG) glutathione, iron, uric acid, and thiobarbituric acid-reactive substances (TBARs) (lipoperoxidation product), as well as knee extensor/flexor muscle torque peak and average power (isokinetic test), were assessed pre- and post-supplementation with Gln or placebo (30 days). Higher plasma D-fructosamine, insulin, and iron levels, and lower strength/power of knee muscles were found pre-supplementation in the NPE group than in the PE group. Post-supplementation, Gln subgroups showed higher levels of GSH, GSSG, and torque peak, besides lower D-fructosamine than pre-supplementation values. Higher muscle average power and plasma uric acid levels were reported in the PE + Gln group, whereas lower insulin levels were found in the NPE + Gln than pre-supplementation values. TBARs levels were diminished post-supplementation in all groups. Gln supplementation, mainly when associated with physical exercises, improves strength and power of knee muscles and glycemia control, besides boosting plasma antioxidant capacity of elderly women.

## 1. Introduction

Aging is a natural process associated with a dynamic and progressive dysfunction of many physiological systems in living organisms [1]. Morphological, functional, biochemical, and psychological alterations result from co-acting variables, such as genetic factors, lifestyle, environmental issues, and diseases [2,3].

According to the document “World Population Ageing 2019—Highlights” (presented by the Department of Economy and Social Affairs of the United Nations Secretariat), by 2050, one in six people in the world will be 65 years of age or older, which will input hard pressure on global health and economy [4]. While developed countries have long lived with a large contingent of elderly people, developing countries began to face this new situation mainly from the 20th to 21st-century transition and beyond [5].

The search for healthy aging has long been the goal for many people worldwide [6]. In this sense, it is essential to point out that healthy aging is vital for the individual to perform his/her daily activities in an independent and safe way [7]. This ability is directly dependent on the efficiency of the neuronal, cardiovascular, and musculoskeletal systems and, on the other hand, is adversely affected by chronic diseases [8]. There is convincing evidence that sedentarism predisposes the individual to higher risks of chronic diseases, age-related comorbidities, and premature death [8].

Regular physical exercises can undoubtedly mitigate the harmful effects of a sedentary lifestyle on people’s health. At this point, it has been highlighted that combined exercise training (associating both aerobic and resistance exercises) is more effective than any other forms of physical exercises performed alone [1]. In older people, combined exercises optimize the acquisition, maintenance, and recovery of the lost physical abilities [1,9].

Beyond the regular practice of physical exercises, efficient nutritional interventions should be applied to minimize the harmful impact of aging [10,11]. Malnutrition due to an inadequate intake of calories or protein plays a role in some of the worst endpoints of aging, e.g., muscle weakness, sarcopenia, frailty, and premature death [12]. Limited access to healthy foods, malabsorption, anorexia, and depressive behavior are among the leading causes of aging malnutrition [12].

A low energy intake may result in the depletion of muscle mass, leading to impaired musculoskeletal system function and physical disability [13]. Age-related skeletal mass loss closely associates with reducing intracellular protein content, especially key metabolic crosslink/anaplerotic amino acids, such as L-glutamine (Gln) [14]. In this sense, supplementation with protein or even isolated L-amino acids can be useful to circumvent this depletion [15]. Although there is no consensus on the ability of Gln to induce muscle hypertrophy [16], some studies reported that supplementation with this non-essential amino acid minimizes the loss of muscle mass [17]. In this respect, our research group reported that Gln supplementation for 15 consecutive days not only increased the expression of signaling factors for protein synthesis, but also reduced the expression of those involved in the protein degradation pathway in soleus muscle of diabetic rats [18]. Corroborating these findings, we also reported lower intracellular levels of Gln associated with increased protein breakdown, whereas oral Gln supplementation attenuates skeletal muscle atrophy induced by fasting [17]. In vitro, Gln improves skeletal myocyte differentiation and prevents myotube apoptosis [19]. Gln also attenuates inflammation and oxidative stress-mediated muscle proteolysis [20].

The information above led us to investigate whether Gln supplementation, associated or not with a regular practice of combined exercise training (CET), could improve glycemia control (as an antidiabetic effect), restrain oxidative stress, and enhance knee muscle strength and power in elderly women.

## 2. Materials and Methods

### 2.1. Subjects and Study Design

**Figure 1:**
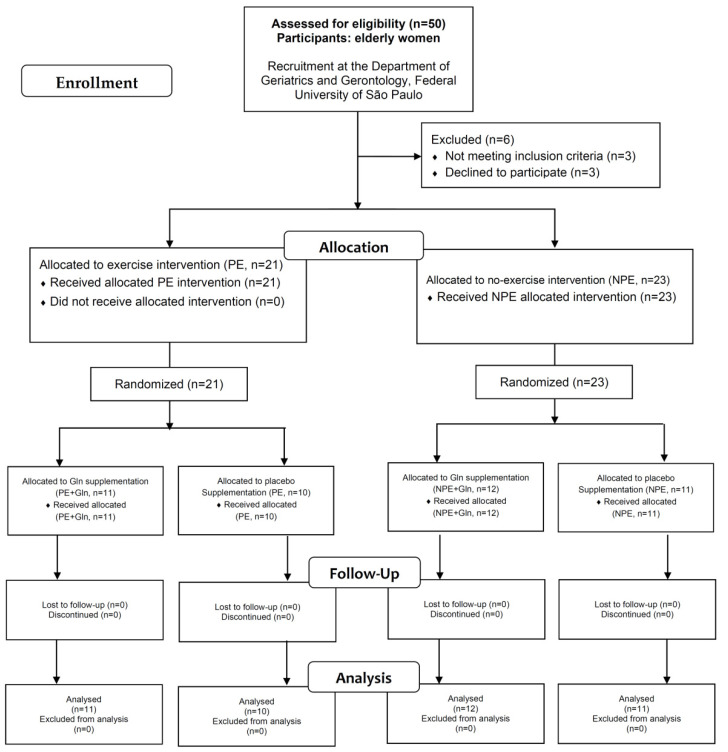
Flowchart of study participants.

This was a pre/post-interventional double-blind randomized study with two major endpoints: (i) to evaluate whether Gln supplementation is able to improve plasma antioxidant capacity when associated with regular exercises (focusing on glutathione metabolism); and (ii) whether Gln supplementation could enhance muscle strength and better control glycemia in exercising elderly women. As shown in the flowchart diagram (Figure 1), 44 elderly women (age between 60 to 80 years, average 69.2 ± 4.5 years) participated voluntarily in this study. All volunteers were recruited from the Primary Health Care Program of the Department of Geriatrics and Gerontology, Medical School, Federal University of São Paulo (UNIFESP), São Paulo, Brazil. The same geriatric physician, coordinator of this Primary Health Care Program at UNIFESP and coauthor of this study, carried out the clinical and physical examinations. The inclusion criteria for volunteers’ selection and recruitment were: elderly women 60 to 85 years old; with clinical and medical authorization to perform the regular program of physical exercises; and with complete registration in the Primary Health Care Program (UNIFESP), including the contact of a responsible person for any emergency. The exclusion criteria were: pre- or current diagnosis of asthma; type-1 diabetes mellitus; neoplastic, renal, or liver diseases; dementia; thrombosis; or angina—diets with more than 4000 cal/day or a protein intake >1.75 g/kg body mass at the time of the study—volunteer taking any antioxidant or multivitamin supplements—use of anti-inflammatory drugs in the last two months. The weekly protein consumption (from main sources: poultry, fish, beef, and pork) of both NPE and PE volunteers is presented as Supplementary Materials.

After exclusion criteria, the volunteers were split into two groups: (i) exercising elderly women (PE, *n* = 21); and non-exercising elderly women (NPE, *n* = 23). Then, each group was subdivided according to L-glutamine (Gln) or placebo supplementation (details ahead), thereby, composing a four-group pre/post-interventional intercrossed study.

Volunteers from the PE group attended the Primary Health Care Program (UNIFESP) for, at least, 24 months, performing their regular physical exercise program at the same place and supervised by the same experienced instructor throughout the study.

Non-exercising volunteers (NPE group) were also attending the Primary Health Care Program (UNIFESP) for appointments and psychological support during identical ≥ 24 months. Although independent and active, they were not involved in any regular exercise program during this period. These volunteers were oriented to maintain their routine during the study.

The Ethics Committee of Cruzeiro do Sul University approved this study (protocol number #213/2014), and it is in agreement with both the Ethical Standards and Declaration of Helsinki. All volunteers were informed of the possible risks involved in the study and all of them signed written consent forms.

### 2.2. Glutamine Supplementation

As aforementioned, PE and NPE volunteers were randomly separated into two subgroups: (i) L-glutamine group (Gln), who ingested 10 g of Gln + 10 g of maltodextrin diluted in 250 mL water (PE + Gln, *n* = 11; NPE + Gln, *n* = 12); and (ii) placebo group (PL), which ingested 20 g of maltodextrin also diluted in 250 mL water (PE, *n* = 10; NPE, *n* = 11), per day. The Gln supplement was purchased from Tongliao Meihua Biological Sci-Tech Co. Ltd. (QiXu, China) and maltodextrin from PR Netto Indústria e Comércio de Alimentos Ltd.a., (São Paulo, SP, Brazil). Supplementation was conducted for 30 days. The experimental design is in Table 1.

It is of utmost importance to clarify that the supplement randomization was carried out following this process: First, we created (computer-generated) a random list of numbers for sequential groups: PE + Gln (*n* = 11), NPE + Gln (*n* = 12), PE (*n* = 10), and NPE (*n* = 11). Each volunteer was then anonymously listed in the experimental groups (in the order as mentioned earlier) according to their recruitment ordering number in the program. After that, the volunteers received a package with 30 sachets of Gln or placebo supplement as designed by the random distribution above. The use of maltodextrin (placebo) was here recommended since it provides the same taste, color, and texture of Gln as a supplement without any visual distinction between them. The volunteers were oriented to ingest the supplement 1× per day, during lunchtime. Dosages of Gln supplement above 30 g/day have been avoided since volunteers have attested stomach discomfort, flatulence, or even more rare, diarrhea under those circumstances (personal communications).

### 2.3. Body Composition

The body composition of volunteers was measured by dual X-ray absorptiometry (DXA—GE Healthcare Lunar, Madison, WI, USA). The DXA scanner was calibrated daily, as instructed by the manufacturer’s guidelines. We used the software (12.3, Lunar DPX, Madison, WI, USA) to estimate body composition.

### 2.4. Determination of Daily Physical Activity

We assessed the daily physical activity of volunteers using the International Physical Activity Questionnaire (IPAQ) according to Craig et al. [21], adapted and validated for the Brazilian population [22]. In agreement with the World Health Organization (WHO), an individual can be considered “active” with a physical activity level above 150 min/week, while levels < 150 min/week classify the subject as “sedentary” [23].

### 2.5. Exercise Program

The exercise regime performed by elderly women from PE-groups involved endurance (aerobic) and resistance (strength training) physical exercises. The combined exercise program followed the guidelines for exercise prescription recommended by the American College of Sports Medicine [1,9] and performed moderately.

Daily exercises were conducted for 60–75 min, three times a week, on non-consecutive days. The aerobic exercises were performed during the first 30 min of sessions, between 60 to 70% of the maximal heart rate reserve, calculated by equation [24]:

HR_max_ = 208 bpm − (0.7 × age), (1)

Aerobic regimes included exercises in step platforms, jump, coordination, and rhythmic movements, all performed in a low impact mode. After the aerobic exercises, the volunteers were submitted to 30–45 min of resistance exercises that involved, at least, five different exercises for different muscle groups: upper and lower limb muscles, abdomen, gluteus, and muscles related to core/postural stabilization, including dorsal and lumbar muscles. All resistance exercises were performed slowly in two series of 10–20 repetitions each, between 50 to 60% of 1-RM (repetition maximum). The resistance exercises compiled different combinations of two muscle groups (described above) and in four consecutive sessions during 30–45 min. Soreness and physical effort were estimated by the post-session Borg Scale, which was also used for monthly weight load adjustments. Monthly cardiac control was also applied for endurance/aerobic adjustments (Polar, FT1, Helsinki, Finland).

### 2.6. Isokinetic Strength Testing

Before the isokinetic testing, the volunteers performed a 5-min warm-up on a cycle ergometer (Cybex Inc., Ronkonkoma, NY, USA) at a resistance level of 25 W, followed by low intensity dynamic stretching exercises for the hamstrings and quadriceps (to avoid stretching influence in strength values). Following the warm-up period, volunteers performed the isokinetic concentric strength test of both lower limbs on a calibrated isokinetic dynamometer (Biodex Medical Systems Inc., Shirley, NY, USA) in a random order. Peak torque (PT) and the average power (AP) of knee flexor and extensor muscles (dominant and non-dominant) in concentric activity were measured. The concentric activity was evaluated at 60°/s and 240°/s. All volunteers completed three submaximal trials before each velocity-test for equipment familiarization and five maximal repetitions to test strength at 60°/s and 240°/s.

### 2.7. Functional Fitness Tests

Functional fitness results were all expressed in seconds in the timed up-and-go test (TUGT), in the 5-times chair stand test (5XST), and in the number of full steps completed in the 2-min step test (2 min step). These tests were specifically recommended for fitness evaluation of older adults [25].

### 2.8. Biochemical Assays

#### 2.8.1. Sample Collection

Blood samples were collected in EDTA-containing Vacutainer^®^ tubes at 8:00 a.m. after a 12 h fast on two different occasions: before (Pre) and 30 days after (Post) supplementation with Gln or placebo. The PE group performed its last exercise training session at least 24 h beforehand. Plasma samples (500 μL) were obtained after centrifugation of blood samples (400× *g*, 10 min) and stored at −80 °C until laboratory analysis.

#### 2.8.2. Determination of Plasma D-Fructosamine and Insulin Concentrations

Plasma insulin concentration was determined by the ELISA technique using a commercial kit (mU/L, Mercodia, Uppsala, Sweden), and the plasma D-fructosamine concentration was determined by a colorimetric commercial kit (μmol/L, Labtest, Minas Gerais, Brazil). All the evaluations were performed to monitor anti-diabetic responses in volunteers, following manufacturers’ instructions.

#### 2.8.3. Determination of Reduced/Oxidized Glutathione (GSH/GSSG)

Reduced (GSH) and oxidized (GSSG) glutathione contents in plasma were determined using colorimetric kits (Bioassay System, Hayward, CA, USA) following the manufacturer’s instructions. The reducing power of plasma was calculated based on the ratio between reduced (GSH) and total glutathione (GSH + GSSG) in the plasma of volunteers [26].

#### 2.8.4. Determination of Iron, Uric Acid, and Lipid Oxidation Indexes

Commercial kits purchased from Bioclin-Quibasa (Belo Horizonte, Brazil) were used to quantify total iron (#K017-1), and uric acid (#K139-1) concentrations. Lipid peroxidation was assayed as thiobarbituric acid-reactive substances (TBARs) in plasma [27]. The concentration of TBARs in plasma was measured after sample treatment with 4% butylated hydroxytoluene (BHT, in ethanol) and further reaction with 0.375% thiobarbituric acid in 0.25 M HCl and 1% Triton X-100 (15 min, at 100 °C). Malondialdehyde equivalents (μmol MDA/mg protein) were calculated based on absorbance at 535 nm against blanks lacking TBA and using 1,1,2,2-tetroxyethylpropane as standard.

### 2.9. Statistical Analysis

All data obtained in this study were initially analyzed for adherence to Gaussian distribution by the Shapiro–Wilk test. Homogeneity of variance was further evaluated by the Levene test. Values with adherence to the normality hypothesis (parametric variables) were presented as mean and standard deviation (x ± SD) and the Student’s *t*-test was used to assess significant differences for the anthropometric, IPAQ, and functional fitness data between (NPE versus PE) groups. In addition, the Chi-square test was used to determine associations between categorical variables in the clinical conditions. The acceptance of the null hypothesis was also tested between pre- and post-supplementation periods in order to evaluate possible skewness or multiple modes from the differences. In this sense, to identify significant differences between the two experimental groups (NPE versus PE), supplemented with L-glutamine or placebo in the pre- and post-supplementation periods, we used a two-way ANOVA test for repeated measures with Student–Newman–Keus post-hoc test in the parametric variables, which were presented as mean and standard error (x ± SE, bars graphic). In addition, Kruskal–Wallis test, combined with the Muller–Dunn post-hoc test, was used to assess the TBARs results since we observed deviation from normality for this parameter (a non-parametric variable, which is presented as a median and interquartile range, box plot graphic).

The effect size (ES) for intragroup analysis was calculated using Cohen’s coefficient and values: (i) between 0.2 and 0.49 indicated a small effect; (ii) between 0.5 and 0.79 indicated a moderate effect; and (iii) higher than 0.8 indicated a large effect [28].

The significance level was set to 5% (*p* < 0.05).

## 3. Results

Table 2 shows anthropometric parameters, clinical conditions, physical activity levels, and performance scores of the functional fitness tests in exercising (PE) and non-exercising (NPE) groups, regardless of Gln supplementation. No differences were found between the groups concerning age or anthropometric characteristics. Only two significant differences were observed regarding clinical conditions: occurrence of arthritis and depression in the NPE group. By IPAQ evaluation, the PE group showed higher physical activity time (+78% and +244% for low and moderate levels, respectively) and also shorter scores (−33%) of sitting time than the NPE group. A significantly lower time to perform TUGT was observed in the PE group than NPE for functional fitness tests performance (−19%; Table 2). No other significant differences were found between the experimental groups. All volunteers were instructed to return the sachets after the supplementation period in order to evaluate their adherence to the supplementation schedule.

**Figure 2:**
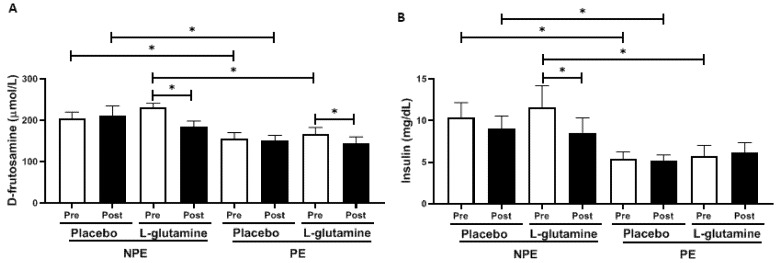
Plasma concentration of (**A**) D-fructosamine (μmol/L) and (**B**) insulin (mg/dL) in non-exercising (NPE) and exercising (PE) older women supplemented with L-glutamine or placebo for 30 days. * *p* < 0.05.

Figure 2 shows D-fructosamine (a) and insulin (b) levels in volunteers before (pre) and after (post) the supplementation period. Regarding placebo volunteers, the intergroup analysis showed that NPE subgroups showed approximately +30% higher D-fructosamine and +92% higher insulin than PE subgroups before (pre) the supplementation period. Similarly, Gln-supplemented volunteers also showed approximately +30% higher D-fructosamine and +107% insulin levels than PE at baseline. In the intragroup analysis, both subgroups supplemented with Gln reduced the D-fructosamine levels (Figure 2A) post-supplementation compared to baseline values (pre): −28% (effect size, ES = 0.93) and −16% (ES = 0.81) for NPE and PE, respectively. However, it is possible to observe in Figure 2B that only the NPE + Gln subgroup presented lower insulin levels (−23%, ES = 0.92) than corresponding values before (pre).

**Figure 3:**
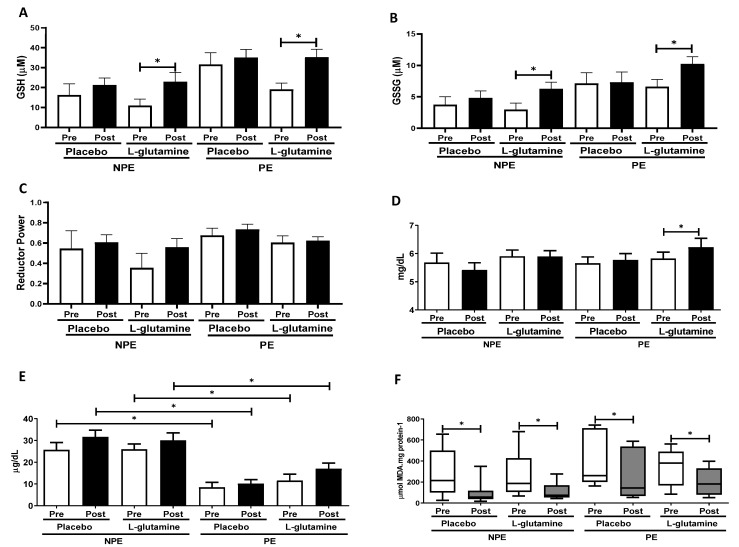
Plasma concentration of: (**A**) reduced glutathione (GSH) (μM), (**B**) oxidized glutathione (GSSG) (μM), (**C**) reductive power (dimensionless), (**D**) uric acid (mg/dL), (**E**) iron ions (mg/dL), and (**F**) thiobarbituric acid-reactive substances (TBARs) (μmol MDA.mg protein^−1^) in non-exercising (NPE) and exercising (PE) older women supplemented with L-glutamine or placebo for 30 days. * *p* < 0.05.

Figure 3A–F shows the oxidative stress indexes before (pre) and after (post) Gln supplementation in NPE and PE groups: reduced (GSH) and oxidized glutathione (GSSG); the reductive power (ratio between GSH and GSSG); and plasma concentrations of uric acid, iron, and TBARs (index of lipoperoxidation). Intergroup analysis did not show any significant differences in GSH or GSSG concentrations between subgroups, but the intragroup analysis revealed that NPE + Gln and PE + Gln samples contain higher concentrations of GSH [respectively, 2.1-fold (ES = 0.92) and 2.0-fold (ES = 1.08); Figure 3A] and GSSG [respectively, +71% (ES = 1.01) and +56% (ES = 1.09); Figure 3B] post-supplementation in comparison to (pre) baseline values. No differences were observed in the reduction power though (Figure 3C). Plasma acid uric levels did not vary significantly between groups, except for a slight increase of 7% (ES = 0.72) observed in the pre/post variation of PE + Gln group (Figure 3D). On the other hand, iron content in plasma was significantly lower in exercising (PE) individuals than in NPE (Figure 3E). Interestingly, in the placebo group, the exercise practice was responsible for an average reduction of almost −68% in plasma iron content (no pre/post effect). A much lower decrease was observed in the PE + Gln group (average of −48%; Figure 3E). Data of lipid peroxidation in plasma (TBARs levels) showed non-normalized distribution within the sample population, and therefore, TBARs levels were presented as box plots (Figure 3F). Pre/post variations were observed for TBARs values in all groups: NPE (−34%, ES = 1.07), NPE + Gln (−45%, ES = 0.99), PE (−58%, ES = 0.51), and PE + Gln (−59%, ES = 0.71; Figure 3F).

**Figure 4:**
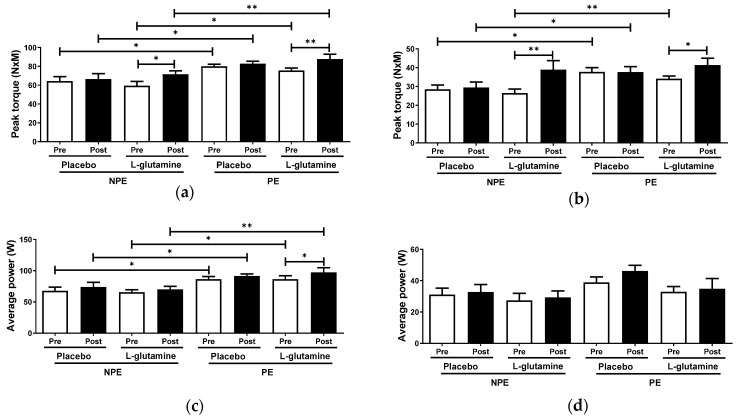
Peak torque and average power of extensor (respectively, **a**,**c**) and flexor knee muscles (respectively, **b**,**d**) in non-exercising (NPE) and exercising (PE) older women supplemented with L-glutamine or placebo for 30 days. * *p* < 0.05; ** *p* < 0.01.

Figure 4 shows concentric peak torque and average power of extensor and flexor knee muscles obtained by isokinetic evaluations of the elderly women groups. Throughout intergroup analysis, PE subgroups showed higher peak torque and power of extensor knee muscle values than NPE subgroups, both pre- (peak torque: PE = +24.5% and PE + Gln = +27.3%; extensor knee average power: PE = +27.5% and PE + Gln = +32%] and post-supplementation (peak torque: PE = +24.6% and PE + Gln = +22.5%; extensor knee average power: PE = +32% and PE + Gln = +39.2%; Figure 4a,b). PE volunteers also showed higher peak torque of flexor knee muscle than NPE before supplementation (PE = +32.7% and PE + Gln = +29.2%; Figure 4c). However, after (post-) supplementation, only placebo PE volunteers sustained the pre-observed increased peak torque (+ 32.7%; Figure 4c). No differences were found on the average power of flexor knee muscle between subgroups, either intergroup or intragroup analysis considered (Figure 4d). The intragroup analysis showed an increase in the peak torque of extensor and flexor knee muscles for both post-Gln supplemented subgroups: +20.6% (ES = 0.91) of extensor and +47.6% (ES = 1.21) of flexor in NPE + Gln, and +16.1% (ES = 1.31) and +21.4% (ES = 0.78), respectively, in PE + Gln subgroups (Figure 4a,c). Figure 4b shows that only the PE + Gln subgroup presented an increase of +12.7% (ES = 0.98) in the average power of extensor knee muscle.

## 4. Discussion

All data considered, we believe that the major findings of this study are: (i) combined exercise regime was able to improve not only glycemia control (as indicated by plasma insulin and D-fructosamine levels), but also restrained prooxidant free iron in the plasma and increased musculoskeletal strength (evaluated by isokinetic tests); and (ii) L-glutamine supplementation had anti-diabetic effects, and improved redox balance and skeletal muscle functions of elderly women, especially in association with an exercise program.

An active lifestyle is undoubtedly vital to hamper the fast progression of degenerative processes associated with aging [29]. The combination of aerobic and resistance exercises has been proven to provide health benefits to the older population by merging gains in muscle strength, neuromuscular control, cardiovascular capacity, endocrine/metabolic rebalances, and cognitive capacity maintenance [30,31]. Interestingly, clinical evidence from our exercising elderly women here (PE subgroup) reinforces that hypothesis, particularly in terms of the reduced use of medicines related to osteoarthritis and depression treatments (Table 2).

Regarding physical conditions, neuromotor activity is often compromised during aging, causing significant reductions in muscle power [32]. Compromised neuromotor connections present both qualitative and quantitative alterations, mainly characterized by a reduction in the conduction speed of the nervous impulse, lower neurotransmitter responsiveness, and progressive reduction in muscle mass, varying with the type of muscle fiber involved (mostly in type II fibers) [33]. Taken together, these alterations promote a decline of elderly physical condition, which is associated with higher risk and occurrence of dangerous falls, as often reported in advanced aging individuals [34]. In this respect, there is a consensus that the regular practice of physical exercises can mitigate the neuromotor alterations and maintain older adults’ functional physical capacity, allowing these subjects to perform their routine activities safely and independently [35].

Many tests are available in the literature to evaluate physical fitness, such as time-up and go test (TUGT), 2-min step in-place test, and 5x sit-to-stand test [36]. Although we did not find differences in the 2-min step in-place and 5x sit-to-stand tests between the elderly women groups, TUGT test revealed that the PE group exhibited a better performance than the NPE group. However, the absolute TUGT values found in both groups (most < 10 s) indicated that all volunteers preserved their functional capacities during the intervention period. The TUGT is an efficient test to assess a whole set of skills, such as balance/strength transitions for sitting–standing positions, stability and speed in walking, and marching direction changes without using compensatory strategies [37]. We examined the benefits of regular combined exercises in older women’s physical capacity, of most, by evaluating the pre/post gains in skeletal muscle strength and power (by isokinetic tests). In healthy elderly people, a progressive loss of muscle power truthfully occurs, in a general way, faster than the strength loss [38].

Although we observed differences between groups in strength of both knee extensor and flexor muscles (peak torque; Figure 4), the average power was unexpectedly different only in the knee extensor muscle. At least part of this fact could be explained by the exercise training program applied, which combined aerobic and resistance training to improve cardiovascular capacity and muscle strength, among other benefits. On the other hand, the combination of aerobic and resistance exercises in the same session may limit the expected gains from each session applied separately [39]. As a hallmark of the aging process, extensor muscles lose around 3.5% of muscle power per year between the ages of 60 and 89, which is associated with alterations in the muscle architecture and reduction of the number and size of type II fibers [40,41]. However, the overall benefits provided by combined exercise programs to elderly people have been extensively reported in the last decades, and it is the best non-pharmacological intervention to avoid most of the neuromuscular and cognitive dysfunctions associated with the aging process [42].

Corroborating this fact, lower plasma levels of insulin and D-fructosamine (a glucose metabolism product) were found in the elderly exercised volunteers (PE) than in non-exercised ones (NPE). Glycemic control is one of the signatures of exercise training in the metabolism, which is particularly important when considering the comorbidities regularly associated with aging, such as metabolic syndrome and type-2 diabetes [43,44,45].

Since it is of utmost importance to control glycemia and to prevent diabetes during aging (for health purposes), there are many studies proposing the use of supplements and phytochemical medicines to accomplish this goal, but aiming the lowest possible side-effects. In this sense, the oral supplementation of L-Glutamine (Gln) has already been demonstrated to control glycemia in diabetic patients via stimulation of glucagon-like peptide 1 (GLP-1) secretion [46]. Additionally, Gln supplementation improves insulin signaling in muscles and liver. Gln is considered the main substrate for hepatic gluconeogenesis, especially in older people [47]. This fact may explain how oral Gln supplementation here (for 30 days) was able to promote a significant reduction of plasma D-fructosamine levels in both PE and NPE old women, together with a significant decrease of insulin levels in NPE. The 30-day placebo supplementation (maltodextrin, a carbohydrate with a high-glycemic index) did not alter the glycemic levels.

Based on recent evidence, at least part of the physiological observations post-Gln supplementation might be related to Gln-mediated adjustments in the redox metabolism [48,49]. Interestingly, Gln was also associated with efficient mitochondrial activity in muscles, whereas Gln depletion impaired myoblast proliferation, differentiation, and the heat-shock response [50]. Since exercises also input additional oxidative challenges (by overproducing reactive oxygen and nitrogen species, ROS/RNS) on activated muscles, cells, tissues, and blood/plasma, it is not surprising to observe inter- and intragroup differences on the monitored redox biomarkers here. Regular exercise causes key (redox) metabolic adaptations in skeletal and cardiac muscles to cope with the oxidative challenges imposed during practices, which are obviously also reflected in plasma. Chronically exercised young and elderly subjects obtain long-term health benefits exactly from the physiological redox stimulus imposed by exercise, demanding proper (and proportional) metabolic, immunological, neuromotor, respiratory, and cardiovascular adaptations [51].

Exercise, any type, causes iron homeostasis disruption, and its extension is apparently dependent on the duration, type (aerobic/resistance), and intensity of exercise, as well as the regularity and previous experience of the tested subjects [52]. Ferrous ions (Fe^2+^) are notorious prooxidants that can catalyze the formation of aggressive ROS/RNS in biological systems, imposing a physiological condition called “oxidative stress” [53]. The exercise-related iron overload is mostly originated from the iron-stocking proteins ferritin and transferrin in the bloodstream or in specific organs, such as the spleen and liver, and also in heme-iron forms from erythrocytes (hemolysis) and contractile muscle fibers (rhabdomyolysis) [54,55]. Therefore, as a product of long-term metabolic adaptations, frequent exercising subjects usually present lower background iron content in plasma, as well as lower peaks of iron release immediately after exercise sessions, compared to sedentary or less-physically active ones [56]. Similar results were also found here, since no apparent effect of Gln was observed on volunteers’ background iron levels, before or after interventions. Background uric acid content in plasma was unaltered among the experimental subgroups, which was not unexpected, since most detectable uric acid variations in plasma were often observed in pre/post analysis of single (intense) exercise bouts [55].

Regarding antioxidant defenses, it has been extensively shown that Gln supplementation restores plasma and muscle Gln levels, leading to adjustments in plasma/muscle redox status (reducing power, or GSH/GSSG ratio), lower indexes of oxidative stress in erythrocytes and skeletal muscle, and even attenuation of pro-inflammatory pathways (e.g., NF-kB), mediated by TNF-α and/or IL-6 [57]. However, it is not clear if Gln supplementation quickly induces GSH biosynthesis in different tissues [58] or activates GSH transport/traffic from stocking organs (like the liver and spleen) [59]. Nevertheless, our results are clearly in agreement with these reports, as Gln supplementation significantly increased GSH levels in both exercised and non-exercised older women here (Figure 3a). Although GSSH levels were apparently unaltered by exercise, the Gln effects were reported in both PE and NPE groups, mainly when observed as pre/post changes (Figure 3b).

According to the literature, the general improvement of antioxidant status and also glycemia control can positively impact muscle function, thus, upgrading its capacity to respond to daily life activities [60]. It is paramount to understand that optimized muscle contraction, obtained during the isokinetic test, requests an adequate energy input and efficient redox balance during the contractile activity. In this sense, the capacity of Gln to serve as a substrate for liver gluconeogenesis could be understood as an additional energy supply for the active muscles during fiber contractions. Such an endergonic effect is obviously extended to sustain muscle contraction during the peak torque test. Concomitantly, the GSH-boost effect of Gln supplementation could also be responsible for an upgrade of plasma and muscle antioxidant defensive systems [48], which could better cope with the oxidative challenged imposed by exercise or intense effort performed during the aforementioned physical/strength tests [49].

Although it was possible to see evidence here for both metabolic (redox balance) and physiological benefits (strength of knee muscles and better glycemic control) induced by 30-day Gln supplementation in exercising elderly women, we could not fully cover the proper molecular mechanism involved. For example, there is a clear link between higher nutritional Gln provision and higher circulating levels of GSH (as well its oxidized form, GSSG). However, we could not determine whether this effect was related to increased GSH biosynthesis or simply enhanced GSH translocation from stocking organs, such as the liver or spleen. In addition, the proper signaling pathways, e.g., Keap1-NRf2, NF-kB, mTOR, etc.—need to be elucidated to explain the results reported here. Nevertheless, the limitations of our work truthfully open new questions and possibilities for further studies.

## 5. Conclusions

In conclusion, we demonstrated, for the first time, that an oral supplementation of L-glutamine for 30 days improves strength and power of knee muscles in association with improved glycemia control and concomitant boost of plasma antioxidant capacity of exercising elderly women.

## Figures and Tables

**Table 1:** Experimental design of the study.

	Pre	Exercise Intervention	Post
	Before supplementation period	30 days (between sampling)	After supplementation period
Blood sampling	X		X
Isokinetic Test	X		X
Gln or placebo supplementation		X	

**Table 2:** Anthropometric parameters, clinical conditions, scores of International Physical Activity Questionnaire (IPAQ), and physical fitness tests in non-exercising (NPE) and exercising (PE) older women pre-supplementation with L-glutamine or placebo for 30 days. (* *p* < 0.05).

Characteristics	NPE (*n* = 23)	PE (*n* = 21)	*p* Value
Age (year)	68.6 ± 4.5	69.8 ± 4.8	>0.05
Height (cm)	155 ± 6.1	155 ± 6.5	>0.05
Weight (kg)	64.1 ± 8.1	59.6 ± 10.5	>0.05
Body mass index (kg/m^2^)	26.5 ± 3.4	24.6 ± 3.1	>0.05
Total body fat (%)	40.3 ± 7.3	43.8 ± 6.8	>0.05
ASM/height (kg/m)	4.4 ± 0.6	6.1 ± 0.3	>0.05
Clinical conditions			
(based on medications use)			
Diabetes mellitus, *n*(%)	3(13)	2(10)	>0.05
Dyslipidemia, *n*(%)	6(26)	6(29)	>0.05
Hypertension, *n*(%)	9(39)	8(38)	>0.05
Coronary heart diseases, *n*(%)	1(4)	1(5)	>0.05
Osteoarthritis, *n*(%)	4(17)	0(0) *	<0.05
Depression, *n*(%)	6(26)	0(0) *	<0.05
Hypothyroidism, *n*(%)	4(17)	3(14)	>0.05
IPAQ			
Low level	497 ± 83	886 ± 175 *	<0.05
Moderate level	413 ± 112	1006 ± 263 *	<0.05
High level	318 ± 70	409 ± 121	>0.05
Sitting time (min/wk)	1745 ± 242	1169 ± 90 *	<0.05
Functional Fitness Tests			
TUGT ^a^ (s)	8.4 ± 1.0	6.8 ± 1.2 *	<0.05
5X ST ^b^ (s)	11.0 ± 2.3	11.0 ± 1.5	>0.05

^a^ TUGT, timed up-and-go test; ^b^ 5XST, 5-times chair stand test.

## Data Availability

Original data is available with the corresponding authors M.P.B. and A.L.L.B. but not archived in databases elsewhere.

## Supplementary Materials

The following are available online at https://www.mdpi.com/2072-6643/13/3/1025/s1.
