# Supplementary material for: L-Glutamine Supplementation Enhances Strength and Power of Knee Muscles and Improves Glycemia Control and Plasma Redox Balance in Exercising Elderly Women
Source: Nutrients. 2021 Mar 22;13(3):1025. doi: 10.3390/nu13031025 (PMC8004646; doi:10.3390/nu13031025)
Supplement: Supplementary file 1 [file nutrients-13-01025-s001.pdf]

Supplementary Material

Tabel. Weekly protein product intake – poultry, fish, beef, pork – by older women volunteering in exercise (PE, n = 21) and non-exercise groups (NPE, n = 23) previous to a 30-day L-glutamine supplementation clinical study. Data were reported based on nutritional questionnaries applied weekly, and scores represent the number of meals including that product per week.

Frequency (times/week)	D[a]				E[b]	N[c]
	1-2×	3-4×	5-6×	+6×		
**PE (n = 21)**						
Poultry	6	7	3	4	1	-
Fish	3	8	2	2	5	1
Beef	6	5	2	3	4	1
Pork	3	4	4	3	6	1
**NPE (n = 23)**						
Poultry	6	7	5	4	1	-
Fish	3	8	1	2	7	2
Beef	6	8	3	2	3	1
Pork	1	7	4	1	7	3

[a] D = daily intake; b E = eventaully; c N = never.
